# Assessing endocrine resistance: monitoring circulating *ESR1* mutations in Irosustat-treated ER positive breast cancer

**DOI:** 10.1007/s10549-025-07857-6

**Published:** 2025-12-09

**Authors:** Karen Page, Luke J. Martinson, Robert K. Hastings, Emmanuel Acheampong, Marc K. Wadsley, Rebecca C. Allsopp, Jin-Li Luo, R. Charles Coombes, Jacqueline A. Shaw, Carlo Palmieri

**Affiliations:** 1 Leicester Cancer Research Centre, Division of Cancer Sciences, University of Leicester, Leicester Royal Infirmary, Robert Kilpatrick Clinical Sciences Building, Leicester, LE2 7LX UK; 2 Nonacus Ltd, Quinton Business Park, Quinton, Birmingham, B32 1AF UK; 3 Department of Surgery and Cancer, Imperial College, Hammersmith Campus, Du Cane Road, London, W12 0NN UK; 4 Department of Molecular and Clinical Cancer Medicine, University of Liverpool, Liverpool, L69 3BX UK

**Keywords:** Liquid biopsy, Circulating tumour DNA, Custom targeted next-generation sequencing, Irusostat, Breast cancer

## Abstract

**Purpose:**

We aimed to investigate the prevalence and spectrum of *ESR1* mutations alongside cell-free DNA (cfDNA) dynamics in patients with estrogen receptor-positive metastatic breast cancer recruited to the phase II IRIS study who had progressed on first-line aromatase inhibitor (AI) therapy and then continued their AI in combination with Irusostat (40 mg), an irreversible steroid sulfatase inhibitor.

**Methods:**

cfDNA was isolated from 96 serial plasma samples from 24 patients, alongside primary tumour DNA (*n* = 16), and analysed by next-generation sequencing using a custom-designed mutation panel on the Illumina NovaSeq platform.

**Results:**

Thirteen of 16 tumour DNA samples harboured at least one somatic mutation across nine genes. Twenty one of the 24 patients (88%) had at least one somatic mutation in cfDNA (248 total mutations across 10 genes). Circulating tumour DNA *ESR1* mutations (ct*ESR1*m) were the most prevalent, present in 16 patients (76%) with both stable (SD) and progressive disease (PD), showing no clear association with disease progression. Eleven patients had polyclonal ct*ESR1*m within the ligand-binding domain, six at baseline, while five harboured a single ct*ESR1*m variant. Five other patients acquired polyclonal mutations over treatment.

**Conclusions:**

Analysis of serial plasma samples revealed highly dynamic ct*ESR1*m during AI treatment and frequent detection of polyclonal ct*ESR1*m in patients (both with SD and PD) recruited to the IRIS study. These findings, albeit in a limited sample size, underscore the challenge of targeting a single *ESR1* mutation and emphasise the need for careful patient selection, specifically those with wild-type *ESR1*, in trials investigating sequential estrogen-lowering therapies.

**Supplementary Information:**

The online version contains supplementary material available at 10.1007/s10549-025-07857-6.

## Introduction

Breast cancer (BC) is the most common cancer in women worldwide, with more than 600,000 people dying annually and despite advances in treatment and detection, these deaths are largely due to metastatic recurrence occurring more than 5 year post-diagnosis [1]. About 75% of BC are estrogen receptor alpha (ERα) positive (ER + ve), this plays a pivotal role in its development and progression as it relies upon estrogen-induced ERα transcriptional activation for growth. ER expression is related to patient age and correlates with lower tumour grade and proliferation, less aneuploidy, less frequent amplification of the c-erbB2 (HER2) oncogene and progesterone receptor (PR) expression [2]. These clinical factors, together with ER expression, guide treatment decisions, and particularly in those patients with metastatic disease, where endocrine therapy remains the most effective option.

Endocrine therapy targets ER by depriving the tumour of estrogen (E2) or by inhibiting ER binding with an agonist. Aromatase inhibitors (AIs), such as anastrozole and letrozole, block aromatase and interfere with conversion of androgens into estrogens, and form the backbone of treatment for ER + ve BC (Supplementary Fig. 1). *ESR1* gene mutations (ct*ESR1*m) that typically occur in the ligand binding domain (LBD) of ERα lead to constitutional activation of the receptor and resistance to therapies [3]. With the advancement of endocrine therapies however, there is increased concern of the potential for resistance [4], and particularly that which is ‘acquired’, as approx. 20% of patients who present with early disease will develop resistance manifested as recurrences either during or after endocrine treatment [5]. This is defined by tumours which typically show a good overall early response, but over the course of therapy become unresponsive [6], compared to de novo resistance, which occurs before treatment with no response to first line endocrine therapies. Potential mechanisms of endocrine resistance include loss of estrogen dependence [7] or inefficiencies of therapies, e.g., due to modulation of signalling cascades [8, 9]. However, more recently, deep-sequencing studies have highlighted the importance of acquired mutations of ER in driving resistance [10], where acquisition of such mutations renders the cancer cells insensitive to AIs and is predicted to reduce sensitivity to anti-estrogens [3, 11].

In addition to managing postmenopausal BC through decreasing circulating levels of oestradiol via inhibition of aromatase [12], the steroid sulphatase (STS) pathway, a second major pathway for estrogen biosynthesis, can also be targeted. Studies have shown increased protein levels of STS in BC are associated with large tumour size, increased risk of recurrence and poorer overall survival [13]; therefore, this alternative pathway is also of significance. Two phase I studies of a first-generation inhibitor of STS, Irusostat (STX64; a tricyclic coumarin sulfamate), showed it to be potent, well-tolerated and caused a significant decrease in serum concentrations of steroids with estrogenic properties [14, 15]. Following this, a phase II combination study (IRIS) was designed to investigate the efficacy and tolerability of Irosustat in postmenopausal women who had progressed on a first-line AI from which they had derived clinical benefit [16]. Patients were followed up each month for 6 and 3 months thereafter, until disease progression or unacceptable toxicity occurred, with the hypothesis that the blockade of STS with Irosustat on the background of continued AI could result in clinical benefit.

In this study, we analysed circulating cell-free DNA (cfDNA) from serial plasma samples of 24 patients enrolled in the IRIS phase II trial collected over a median of 3 months (range 1–18 months) on the study. Using a custom-designed targeted next-generation sequencing panel relevant to metastatic breast cancer, we profiled mutational hotspots, focussing on *ESR1*, to characterise the ctDNA genomic landscape in patients progressing on first line AI who were then continued on their AI therapy combined with the steroid sulfatase (STS) inhibitor Irosustat. Our objectives were to quantify circulating tumour DNA (ctDNA) levels, assess ctDNA dynamics, and explore how ctDNA *ESR1* mutations (ct*ESR1*m) may influence treatment response, as their impact on Irosustat efficacy remains unclear.

## Materials and methods

### Patients and samples

The IRIS study (ClinicalTrials.gov NCT0178 5992) was a multicentre, open label phase II trial performed in nine academic medical centres in the United Kingdom conducted in accordance with Good Clinical Practice Guidelines and the Declaration of Helsinki. Ethical approval was given by the Riverside Research Ethics Committee (an Independent Ethics Committee; reference 12/LO/0477), and approved by the United Kingdom Medicines and Healthcare Products Regulatory Agency (EudraCT: 2011–005680-25). All participants gave written informed consent prior to participation and were over 18 years of age.

**Fig. 1:**
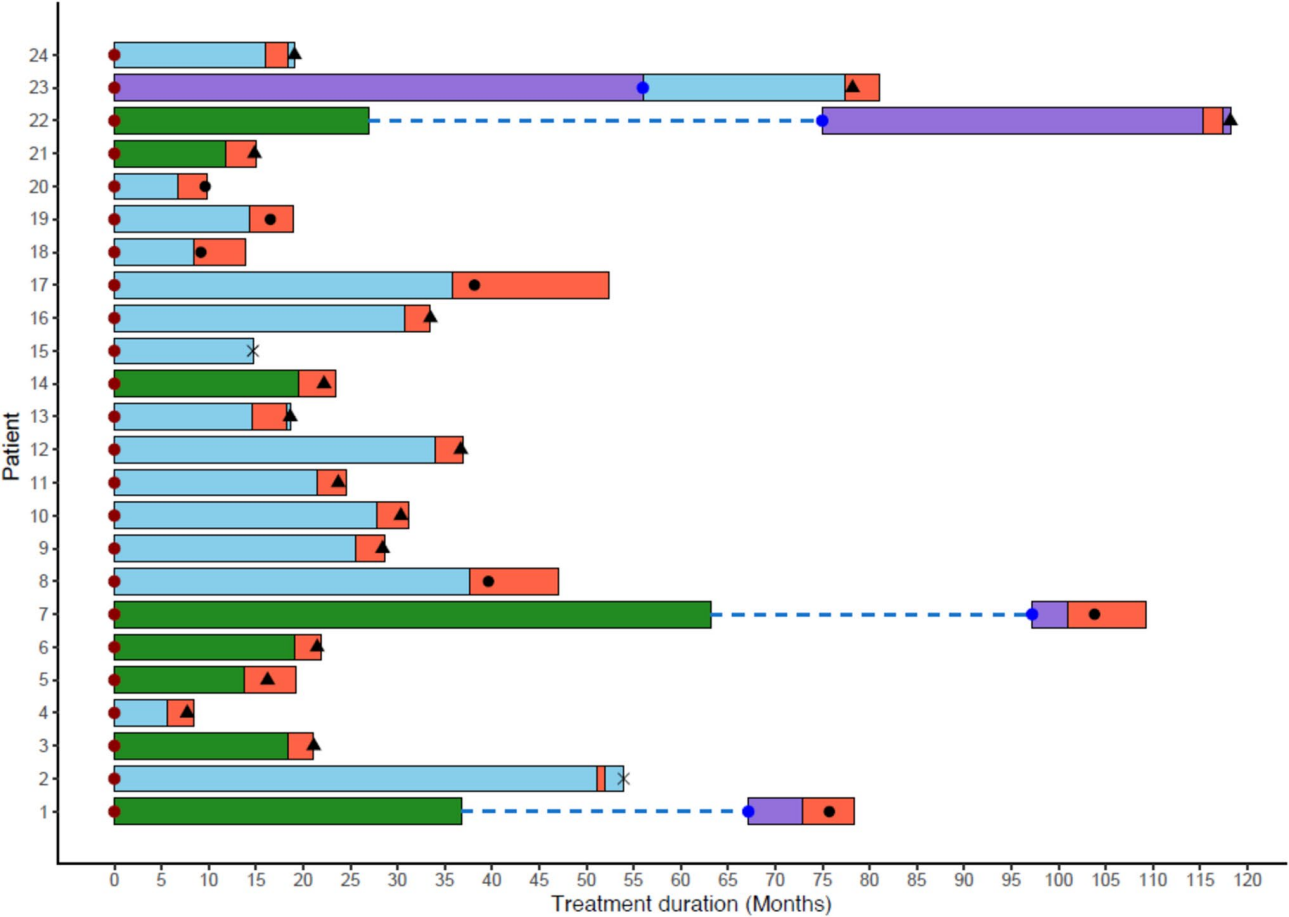
Swimmer plot depicting AI therapy duration and radiological evaluations of patients enrolled in IRIS study, illustrating individual patient treatment durations. Start and end dates of treatment are indicated by the left and right edges of each bar, respectively (months; anastrozole (green), exemestane (purple), letrozole (blue) and Irosustat (red)). Radiological evaluations are shown by either a black triangle (progressive disease), black circle (stable disease) or a x (unknown). The number of lines of AI therapy are represented by coloured circles on the horizontal bars (1 (purple) and 2 (blue)), with dotted lines representing a break in treatment. All blood samples were taken, while patients were receiving AI plus Irosustat

Women were eligible if they were postmenopausal, with histologically confirmed ER + ve, HER2 -ve inoperable locally advanced or metastatic BC, where ER positivity was based on local laboratory assessment (Supplementary Table 1) and had developed progressive disease during first-line AI therapy for recurrent ER + BC; see Fig. 1 for visual representation of integrated treatment timelines and radiological evaluations. Eligible patients also had to have derived clinical benefit, defined as a documented objective response at any point or disease stabilisation (SD) for at least 6 months, from their first-line AI treatment. The disease had to be measurable by CT/MRI scan according to RECIST v1.1. Patients were monitored by serial blood sampling over a period of 1–18 months along with clinical evaluations. 8 ml blood was taken into K2 EDTA tubes (BD Biosciences) and processed to plasma within 2 h of collection for extraction of cfDNA.

### Extraction and quantitation of DNA

Total cfDNA was isolated from 115 serially collected blood plasma samples from 24 patients, using 4 ml blood plasma with the MagMAX™ Cell-free DNA Isolation Kit (Thermo Fisher Scientific) as described previously [17, 18]. Matching formalin-fixed paraffin-embedded (FFPE) tumour tissue was available for 16 of the 24 patients; 15 were derived from the primary tumour, and one from a metastatic relapse. Tumour DNA was isolated from FFPE tissue blocks using the GeneRead™ Kit (Qiagen), as described previously [18]. Quantitation and quality checks for cfDNA and tumour DNA were performed using the Agilent Tapestation cell-free DNA Screentape (Agilent) and Qubit™ dsDNA HS Assay kit (Thermo Fisher Scientific) respectively, according to manufacturer’s instructions.

### Targeted next generation sequencing

A custom next-generation sequencing panel targeting 397 hotspot mutations across 19 genes (Supplementary Table 2) was developed by informaticians using a bespoke assay design pipeline (Nonacus Ltd). A total of 96 libraries were prepared from plasma DNA across 24 patients (median input: 50 ng). using this and the Cell3™ Target kit (Nonacus). Libraries were pooled equal amounts (*n* = 8) and hybridised with biotin-labelled DNA probes to enrich for the targeted regions. In addition, 16 libraries were prepared from tumour FFPE DNA (median input: 55 ng), pooled together, and similarly enriched. All final captured library pools were sequenced on the Illumina NovaSeq platform. The custom Illumina-based targeted NGS panel achieved a validated limit of detection down to 0.3% variant allele frequency (VAF), enabled by unique molecular identifiers for error suppression.

### Bioinformatic analysis of raw sequencing data

FASTQ files were processed using Nonacus' custom research tumour-only pipelines, which incorporate Sentieon tools (v202112.06) for alignment, variant calling, and quality control [19], FASTP (v0.23.4) for adapter and quality trimming [20]), and the Ensembl Variant Effect Predictor (VEP) (v108.2) for variant annotation [21]. Two workflows were used depending on sample type.

For FFPE samples, raw FASTQ files were trimmed using FASTP [20] then aligned directly to the GRCh38 reference genome using sentieon bwa mem. Reads were sorted, indexed, and recalibrated using QualCal [19]. Somatic variants were called using TNhaplotyper2 in tumour only mode, followed by orientation bias and contamination correction using OrientationBias and ContaminationModel, as in the cfDNA workflow. UMI-specific steps were omitted. Variant refinement was carried out using TNfilter, with settings appropriate for FFPE data [19], with a 5% VAF cutoff included to represent threshold used in clinical diagnostic settings [22].

For cfDNA samples, raw FASTQ files containing UMIs were first processed using sentieon umi extract, and consensus reads were generated using Sentieon’s UMI consensus module. The resulting consensus FASTQ files were then trimmed using FASTP [20] to remove adapters and low-quality bases. Trimmed consensus reads were aligned to the GRCh38 reference genome using sentieon bwa mem, followed by sorting, indexing, and Base Quality Score Recalibration (BQSR) using QualCal [19].

For cfDNA analysis, somatic variants were called with TNhaplotyper2 [19] using conservative settings optimized for low allele frequencies (–default_af 5e-8, –min_tumor_lod 3, –min_normal_lod 2.2), alongside a panel of normals, germline reference, and curated hotspot set to improve sensitivity while controlling false positives. Variant calls were further refined with OrientationBias and ContaminationModel [19], then filtered with TNfilter (–f_score_beta 1.5, –max_event_count 50, –min_tumor_af 0.001, –max_alt_count 4) to suppress sequencing artifacts and contamination noise, with the aim of maximising sensitivity and enriching for true low-frequency cfDNA variants and further filtered using BCFtools (v1.16) [23].

In both workflows, variants were annotated using the Ensembl Variant Effect Predictor (VEP, v108.2) [21] run in offline mode with cache version 108. Annotated VCFs were compressed and converted to MAF format using vcf2maf (v1.6.21) [24] for downstream analysis.

### Statistical analysis

The Mann–Whitney non-parametric statistical test was used to compare cfDNA yields in patients with stable and progressive disease; Spearman’s rank correlation coefficient was used to investigate the correlation between disease status and ct*ESR1*m variant allele fraction (VAF). These analyses were carried out using GraphPad Prism v10.2.3 software. All *P* values were two-sided and those < 0.05 were considered statistically significant.

## Results

A total of 24 patients with ER + , HER2-ve, inoperable locally advanced or metastatic BC, who were progressing on first line AI were enrolled into the IRIS phase II trial. The mean patient age was 56 years (range: 31–76). All patients received the STS inhibitor Irosustat in combination with a first-line AI: exemestane (*n* = 3), letrozole (*n* = 16), or anastrozole (*n* = 5; Fig. 1; Supplementary Table 1). At the time of Irosustat addition, 7 patients exhibited stable disease (SD), 15 showed progressive disease (PD) and two were unknown. Longitudinal plasma cfDNA samples were collected over a median of 3 months (range 1 to 18; average 4 samples per patients, total 96 across 24 patients). Matched FFPE primary tumour tissue was available for 16 of the patients. All 96 plasma cfDNA samples and 16 tumour DNA samples were successfully sequenced using a semi-automated, standardised workflow under good clinical laboratory practice (GCLP). The median cfDNA concentration in plasma was 24.6 ng/mL (range: 5.1–948.2 ng/mL), with no statistically significant difference in cfDNA levels between progressive and stable disease observed (*P* = 0.0786, Mann–Whitney *U* test); however, the limited sample size precludes firm conclusions.

### Mutational landscape in primary tumours

Applying a cut off 5% VAF in the primary tumour FFPE DNA, targeted next-generation sequencing (NGS) identified 17 somatic missense mutations across 9 genes (*CDH1*, *ERBB3*, *ESR1*, *GATA3*, *KMT2C*, *NF1*, *PIK3CA*, *MAP3K1*, and *TP53*) in tumours from 13/16 patients. Each of the 13 tumours harboured at least one somatic mutation (Supplementary Fig. 2; Supplementary Table 4), with *PIK3CA* the most frequently mutated gene, (5 mutations in 5/13 tumours; 38%). *ESR1* and *TP53* mutations were each detected in 2 tumours (31%). Among 11 patients with PD, ct*ESR1*m were detected in 2 patients (18%); one in metastatic tissue and one in primary tumour tissue. *PIK3CA* mutations were identified in 5 tumours.

## *Circulating tumour DNA profiles and ctESR1*m* resistance dynamics*

**Fig. 2:**
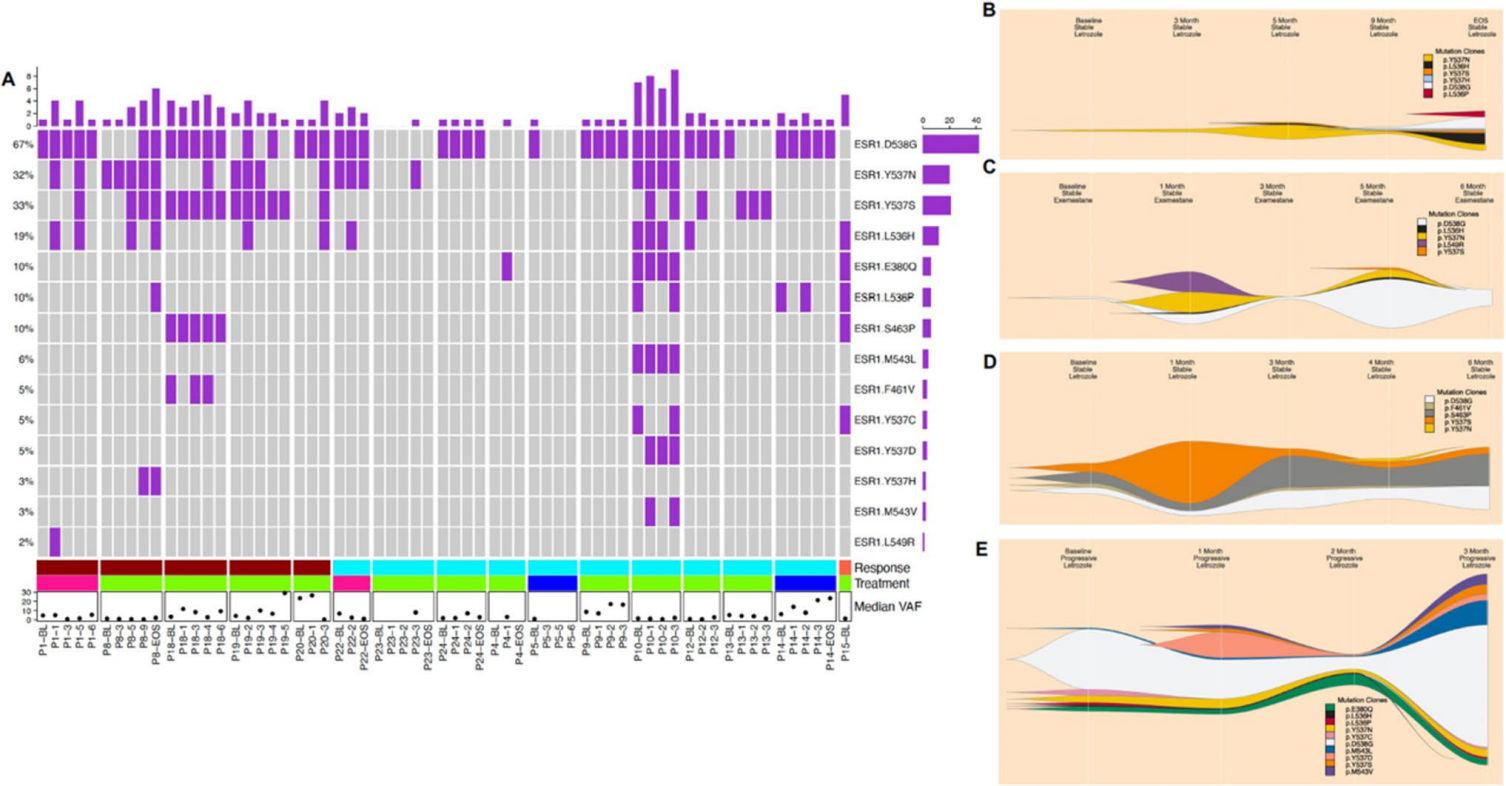
ctDNA *ESR1* dynamics in patients treated with AI and Irosustat, enrolled in the IRIS study (*n* = 24). **A** Oncoprint illustrating the *ESR1* mutational landscape in cfDNA from patients (*n* = 24) treated with different AI therapies (missense mutations (purple), anastrozole (blue), exemestane (pink) and letrozole (green) and; with either stable (*n* = 7; deep pink) or progressive disease (*n* = 15; turquoise) and unknown disease stage (*n* = 1; orange). P indicates patient number followed by either BL (baseline), numbers indicating timepoints in months or EOS (end of study). **B–D** Fish plots depicting ct*ESR1*m dynamics for four individual patients (**B** Patient 8, **C** Patient 1, **D** Patient 18 and **E** Patient 10)

In blood plasma, *ESR1* was the most frequently mutated gene in cfDNA, detected in 16 of 21 ctDNA-positive patients (76%) and accounted for 131 of 248 mutations (53%) across all serial timepoints (Fig. 2; Supplementary Figs. 4, 5 and 6). Common mutations present in both tumour and plasma samples were predominantly in *TP53* and *PIK3CA* (Supplementary Table 6); however, one *ESR1* mutation (p.D538G, 70.8% VAF) was also detected in the baseline plasma sample matched to one patient (P5, 0.75% VAF).

At study entry (baseline sample), 14 out of 16 patients had detectable ct*ESR1*m, with 6 of these cases exhibiting polyclonal mutations (examples are given in Fig. 2B–E); and seven patients showing ct*ESR1*m evolving over time. In total, 11 of 16 patients (69%) had polyclonal mutations located in the ligand-binding domain (LBD), including p.D538G, p.Y537N/S/C, p.L536H/Q/P/R, and p.S463P (Fig. 2; Supplementary Figs. 4, 5 and 6). Of these, 6 patients had polyclonal ct*ESR1*m at baseline, while 5 had single variants. The most prevalent mutation observed was p.D538G (32% of all ct*ESR1*m events).

Correlation analysis revealed no significant association between treatment response and either ct*ESR1*m VAF (*P* = 0.347, *r* = 0.087) or ct*ESR1*m presence/absence (*P* = 0.432, *r* = −0.168; Spearman’s rank correlation). Concerning the 10 genes with mutations, a total of 248 mutations were identified (Fig. 2A and Supplementary Fig. 3).

## Discussion

Analysis of primary tumour DNA from patients progressing on AI therapy and subsequently treated with AI plus Irosustat identified somatic mutations in key BC associated genes, most notably *ESR1*, *PIK3CA*, and *TP53.*

In blood plasma, ct*ESR1*m were the most common prevalent, with 53% of patients exhibiting one or more mutations, with 88% located in the LBD of ERα, aligning with previous studies that observed ct*ESR1*m located in the LBD [3, 11, 23]. Mutations withing the LBD are known to mimic estrogen binding, driving ligand-independent ER activation and resistance to AIs.

Here, ct*ESR1*m were highly dynamic during AI therapy and following the addition of Irosustat, with evidence of frequent polyclonality and fluctuations in VAF over time. This enriched polyclonality is similar to that reported in the SoFEA trial, which established ct*ESR1*m as a clinically relevant mechanism of acquired resistance to AI; [25]). Where polyclonality was common (49%) here, it is most likely a result of the prolonged exposure to AI treatment, although it may also be due, in part, to the limited sample size. The high frequency of polyclonal ct*ESR1*m located in the LBD highlights the molecular heterogeneity of endocrine resistance in advanced ER-positive BC, making it difficult to target with a single endocrine therapy. Such dynamics likely reflect selective pressure from therapy and convergent evolution towards ligand-independent ERα activation [3].

*ESR1*m in primary tumours are extremely rare, they are detected in fewer than 1% of treatment-naïve cases, as demonstrated in the TCGA Breast Cancer study, which analysed over 800 primary breast tumours [24]. Here, a single *ESR1*m (p.R394C) was identified in the primary tumour tissue but was absent from matched cfDNA. This discrepancy highlights both the technical limitations of NGS and the dynamic nature of tumour evolution under treatment pressure. Notably, p.R394C is located outside the LBD, where most activating, resistance-associated *ESR1*m are found and its absence in cfDNA (and location outside the LBD) suggest it may not be a driver of AI resistance and could represent a passenger mutation or a potential sequencing artefact.

Although the earlier IPET study reported a significant reduction in the proliferation marker Ki67 after just 2 weeks of Irosustat treatment [24], our findings indicate that patients harbouring ct*ESR1*m at treatment initiation (14 of 24 patients; Fig. 1B) are unlikely to derive substantial benefit from the addition of Irosustat, as ligand-independent signalling may undermine its efficacy, particularly when used in combination with AIs in patients who already exhibit endocrine resistance. Therefore, retrospective analysis of this cohort underscores the importance of selecting patients with wild-type *ESR1* for future studies involving sequential estrogen-lowering therapies.

Finally, data from two recent clinical trials investigating the efficacy of oral selective estrogen receptor degraders (SERDs) in ct*ESR1*m-positive patients with advanced breast cancer suggest a promising alternative to Irosustat. The EMERALD trial demonstrated that elacestrant, the first approved oral SERD, significantly improved progression-free survival (PFS) in patients with *ESR1*-mutant tumours who had received two prior lines of AI-based endocrine therapy [26]**.** Similarly, the SERENA-6 trial showed that switching to camizestrant plus a CDK4/6 inhibitor upon detection of ct*ESR1*m by ctDNA testing prolonged PFS compared with continuing AI plus CDK4/6 inhibition [26]. Notably, the switch in therapy was guided by the emergence of ct*ESR1*m detected via ctDNA testing, prior to clinical or radiologic progression.

These findings, albeit in a study of limited sample size, emphasise the value of longitudinal liquid biopsy profiling to capture resistance mechanisms that may not be detectable in archival primary tumour tissue. Taken together, these exploratory findings underscore that ct*ESR1*m are dynamic, frequently polyclonal, and fluctuate in VAF during treatment, and demonstrate the potential of ctDNA as a real-time monitoring tool to guide therapeutic decisions while illustrating the challenge of targeting a single *ESR1* variant in patients progressing on AI. Although the sample size prevents firm conclusions, our data supports the potential use of ctDNA as a real-time biomarker for monitoring resistance and informing adaptive treatment strategies, particularly in sequential endocrine therapy, where early identification of resistance-associated mutations may guide timely treatment modification. Moreover, they highlight the clinical value of oral SERDs in overcoming ct*ESR1*m-mediated resistance, compared with limited efficacy of sequential estrogen-lowering strategies, such as Irosustat. This is especially relevant in light of the recent ASCO recommendations supporting routine ct*ESR1*m testing in patients with metastatic ER + ve, HER2-ve BC progressing on hormonal therapy, supported in the UK with ct*ESR1*m being added to the cancer test directory.

## Supplementary Information

Below is the link to the electronic supplementary material.

Supplementary file1 (TIF 93 KB)—Supplementary Figure 1. Estrogen biosynthesis pathways and targets of endocrine therapies.

Supplementary file2 (TIF 253 KB)—Supplementary Figure 2. Oncoprint illustrating all (missense mutations (purple) or truncated (green)) mutations detected in FFPE tumour DNA from BC patients (n=16); with either stable (n=5; deep pink) or progressive disease (n=9; turquoise) and unknown disease stage (n=1; orange). P indicates patient number; cancer type is indicated (infiltrating ductal carcinoma, pink and unknown, blue).

Supplementary file3 (TIF 213 KB)—Supplementary Figure 3. Oncoprint illustrating the mutational landscape in cfDNA from patients (n=24) treated with different AI therapies (missense mutations (purple), anastrozole (blue), exemestane (pink) and letrozole (green) and; with either stable (n=7; deep pink) or progressive disease (n=15; turquoise) and unknown disease stage (n=1; orange). P indicates patient number followed by either BL (baseline), numbers indicating timepoints in months or EOS (end of study).

Supplementary file4 (TIF 139 KB)—Supplementary Figure 4. CtDNA dynamics and cfDNA levels over time in patients with stable disease with at least two ctDNA positive timepoints. Dotted line indicates mutation also detected in tumour DNA. A) P1; B) P5; C) P8; D) P18; E) P19 and F) P20.

Supplementary file5 (TIF 212 KB)—Supplementary Figure 5. CtDNA dynamics and cfDNA levels over time in patients with progressive disease with at least two ctDNA positive timepoints. Dotted line indicates mutation also detected in tumour DNA. A) P4; B) P5; C) P6; D) P9; E) P10; F) P12; G) P13; H) P14; I) P16; J) P21; K) P22; L) P23 and M) P24.

Supplementary file6 (TIF 426 KB)—Supplementary Figure 6. Fish plots representing ct*ESR1*m dynamics A) P19 B) P22 C) P9 D) P20 E) P13 F) P14 G) P12 and H) P24.

Supplementary file7 (XLSX 53 KB)

## Data Availability

Data is provided within the manuscript or supplementary information files.
