# Supplementary figures and images for: Assessing endocrine resistance: monitoring circulating ESR1 mutations in Irosustat-treated ER positive breast cancer
Source: Breast Cancer Res Treat. 2025 Dec 9;215(1):34. doi: 10.1007/s10549-025-07857-6 (PMC12689781; doi:10.1007/s10549-025-07857-6)

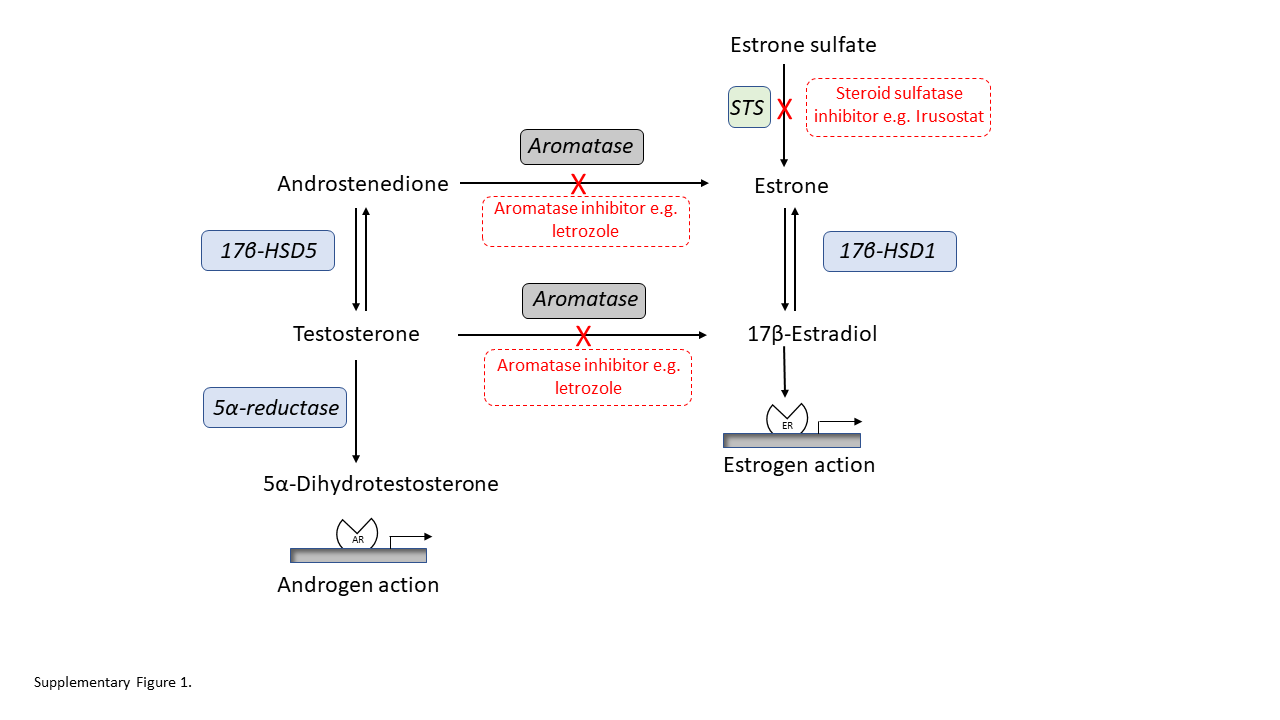

Supplement: Supplementary file 1 — Supplementary file1 (TIF 93 KB)—Supplementary Figure 1. Estrogen biosynthesis pathways and targets of endocrine therapies. [file 10549_2025_7857_MOESM1_ESM.tif]

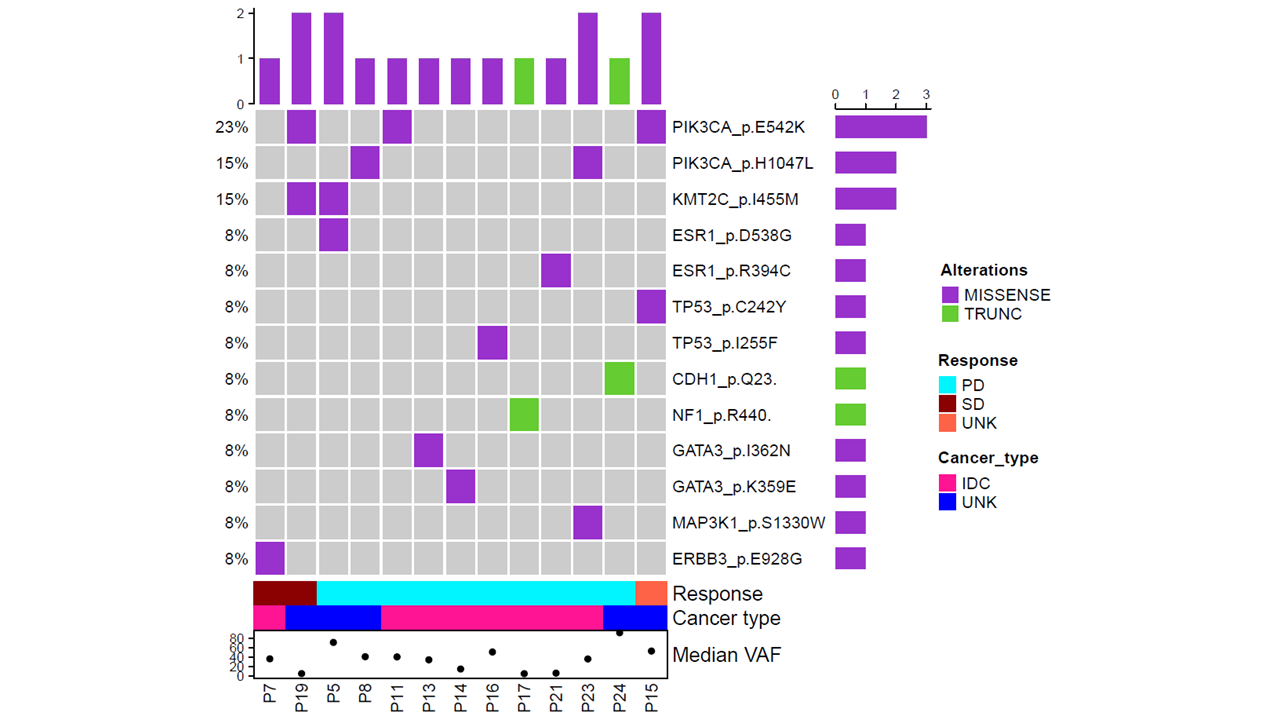

Supplement: Supplementary file 2 — Supplementary file2 (TIF 253 KB)—Supplementary Figure 2. Oncoprint illustrating all (missense mutations (purple) or truncated (green)) mutations detected in FFPE tumour DNA from BC patients (n=16); with either stable (n=5; deep pink) or progressive disease (n=9; turquoise) and unknown disease stage (n=1; orange). P indicates patient number; cancer type is indicated (infiltrating ductal carcinoma, pink and unknown, blue). [file 10549_2025_7857_MOESM2_ESM.tif]

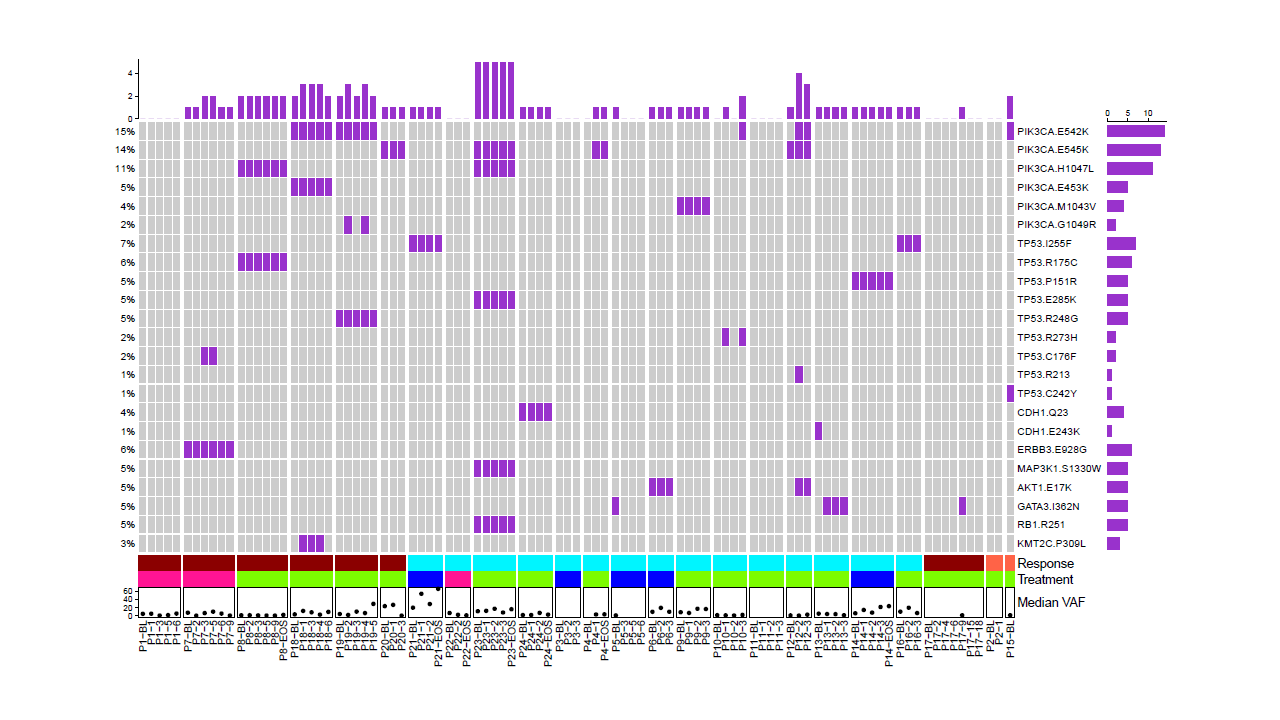

Supplement: Supplementary file 3 — Supplementary file3 (TIF 213 KB)—Supplementary Figure 3. Oncoprint illustrating the mutational landscape in cfDNA from patients (n=24) treated with different AI therapies (missense mutations (purple), anastrozole (blue), exemestane (pink) and letrozole (green) and; with either stable (n=7; deep pink) or progressive disease (n=15; turquoise) and unknown disease stage (n=1; orange). P indicates patient number followed by either BL (baseline), numbers indicating timepoints in months or EOS (end of study). [file 10549_2025_7857_MOESM3_ESM.tif]

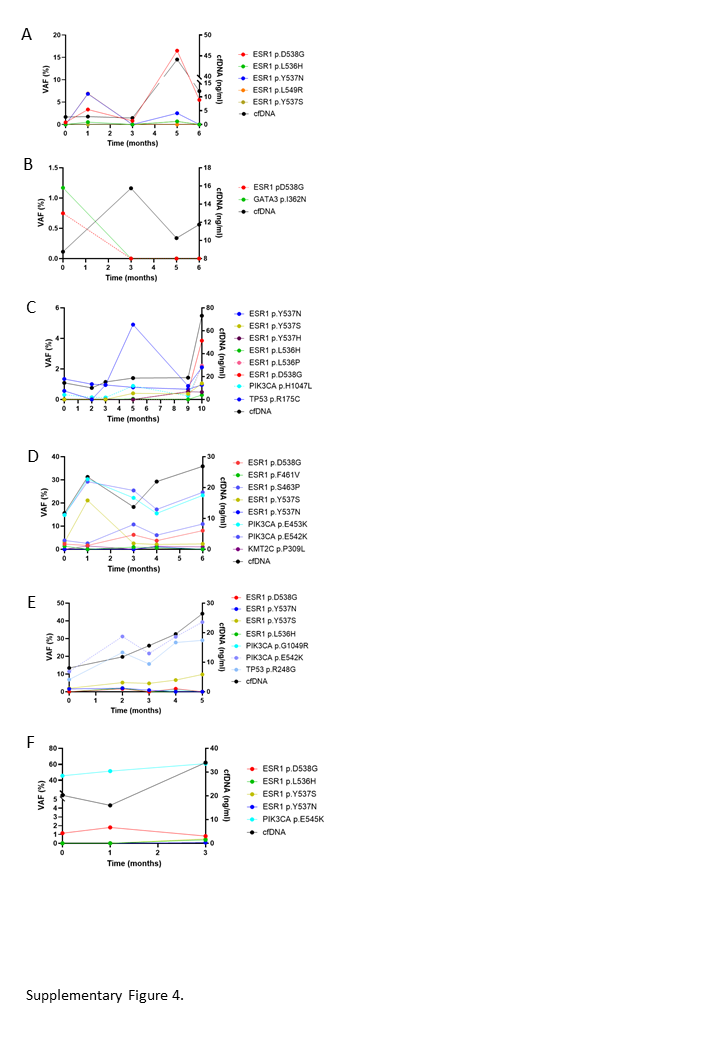

Supplement: Supplementary file 4 — Supplementary file4 (TIF 139 KB)—Supplementary Figure 4. CtDNA dynamics and cfDNA levels over time in patients with stable disease with at least two ctDNA positive timepoints. Dotted line indicates mutation also detected in tumour DNA. A) P1; B) P5; C) P8; D) P18; E) P19 and F) P20. [file 10549_2025_7857_MOESM4_ESM.tif]

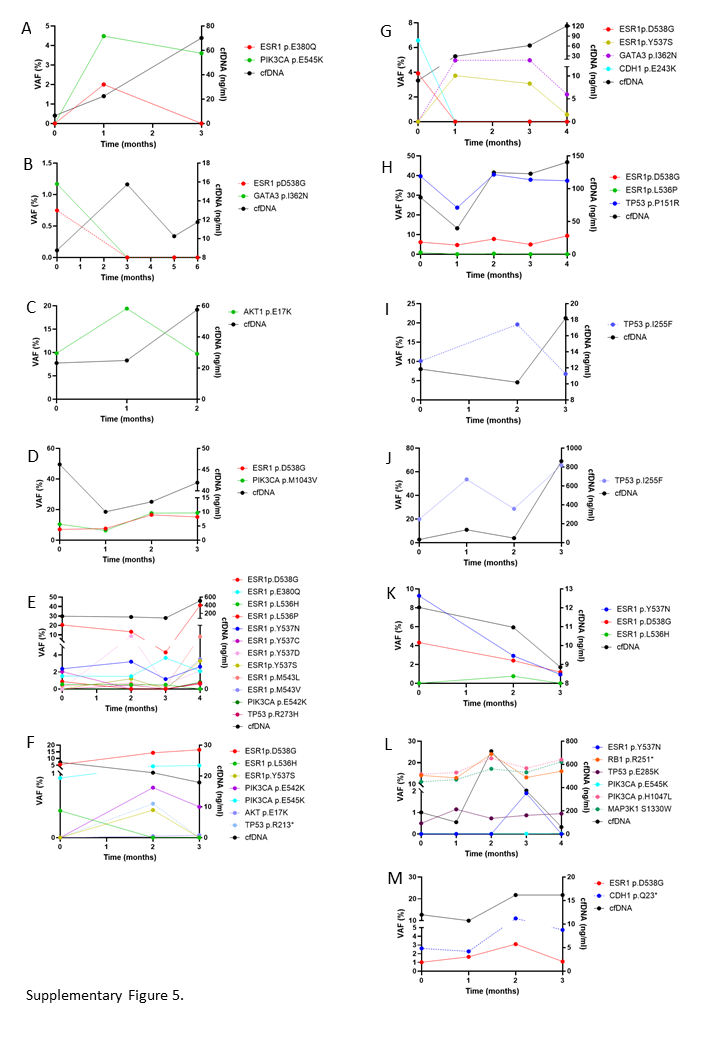

Supplement: Supplementary file 5 — Supplementary file5 (TIF 212 KB)—Supplementary Figure 5. CtDNA dynamics and cfDNA levels over time in patients with progressive disease with at least two ctDNA positive timepoints. Dotted line indicates mutation also detected in tumour DNA. A) P4; B) P5; C) P6; D) P9; E) P10; F) P12; G) P13; H) P14; I) P16; J) P21; K) P22; L) P23 and M) P24. [file 10549_2025_7857_MOESM5_ESM.tif]

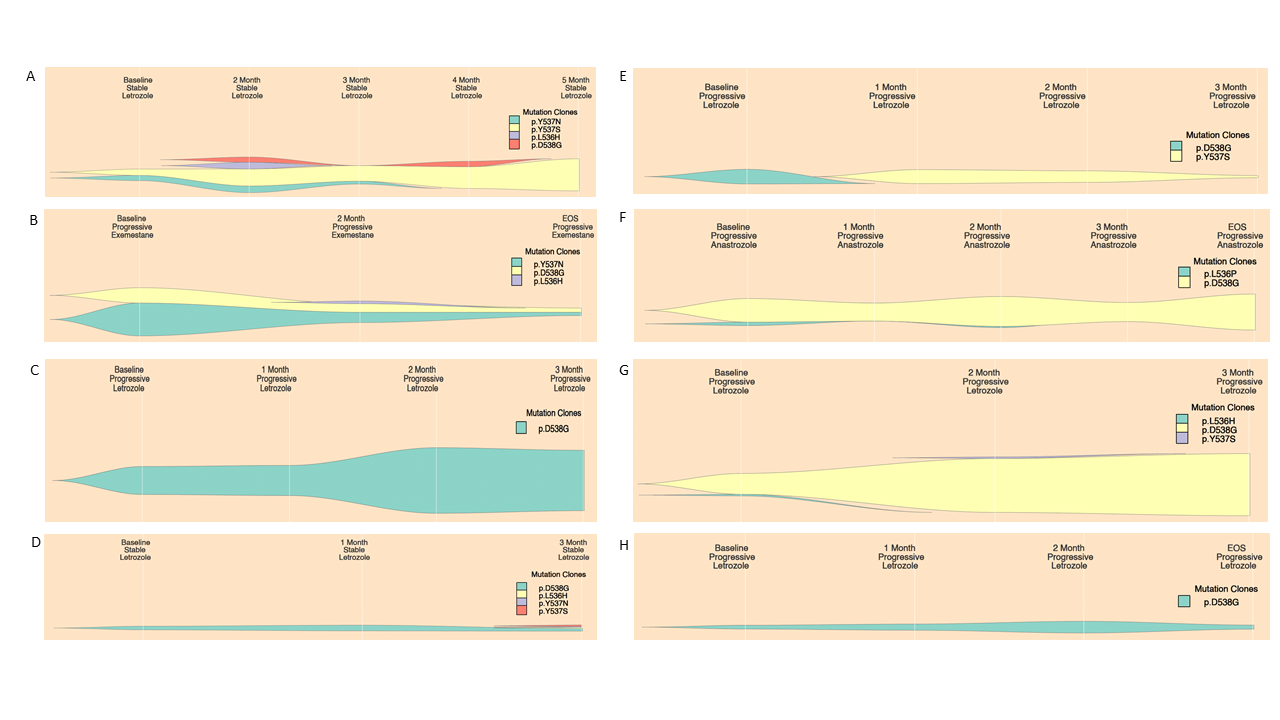

Supplement: Supplementary file 6 — Supplementary file6 (TIF 426 KB)—Supplementary Figure 6. Fish plots representing ctESR1m dynamics A) P19 B) P22 C) P9 D) P20 E) P13 F) P14 G) P12 and H) P24. [file 10549_2025_7857_MOESM6_ESM.tif]
